# Whole Genome DNA Methylation Profiling of D2 Medium Spiny Neurons in Mouse Nucleus Accumbens Using Two Independent Library Preparation Methods

**DOI:** 10.3390/genes13020306

**Published:** 2022-02-06

**Authors:** Yuxiang Li, Haiyang Xu, Javed M. Chitaman, Jian Feng

**Affiliations:** Department of Biological Science, Program in Neuroscience, Florida State University, Tallahassee, FL 32306, USA; yli@bio.fsu.edu (Y.L.); hxu@bio.fsu.edu (H.X.); jchitaman@neuro.fsu.edu (J.M.C.)

**Keywords:** DNA methylation, methylome, DRD2, medium spiny neuron, nucleus accumbens, mouse

## Abstract

DNA methylation plays essential roles in various cellular processes. Next-generation sequencing has enabled us to study the functional implication of DNA methylation across the whole genome. However, this approach usually requires a substantial amount of genomic DNA, which limits its application to defined cell types within a discrete brain region. Here, we applied two separate protocols, Accel-NGS Methyl-Seq (AM-seq) and Enzymatic Methyl-seq (EM-seq), to profile the methylome of D2 dopamine receptor-expressing medium spiny neurons (D2-MSNs) in mouse nucleus accumbens (NAc). Using 40 ng DNA extracted from FACS-isolated D2-MSNs, we found that both methods yielded comparably high-quality methylome data. Additionally, we identified numerous unmethylated regions (UMRs) as cell type-specific regulatory regions. By comparing the NAc D2-MSN methylome with the published methylomes of mouse prefrontal cortex excitatory neurons and neural progenitor cells (NPCs), we identified numerous differentially methylated CpG and non-CpG regions. Our study not only presents a comparison of these two low-input DNA whole genome methylation profiling protocols, but also provides a resource of DNA methylome of mouse accumbal D2-MSNs, a neuron type that has critical roles in addiction and other neuropsychiatric disorders.

## 1. Introduction

DNA methylation is an epigenetic mechanism that plays important roles in gene regulation and genome stability [1]. In mammals, DNA cytosine methylation is vital for development and cell fate commitment, cell identity maintenance, and various other cellular functions [2,3,4,5,6,7,8,9]. DNA cytosine methylation is deposited by a group of DNA methyltransferase enzymes (DNMTs) [10] and can be oxidized by ten-eleven translocation (TET) methylcytosine dioxygenases that may lead to DNA demethylation [6,11].

To study DNA methylation, several methodologies have been developed. For example, methylation-sensitive restriction enzymes are used to infer the methylation status of genomic loci based on their differential enzymatic cutting activity at DNA motifs containing methylated or unmethylated cytosines [12]. In contrast, antibodies against methylated cytosines have also been developed to examine methylation status by immunostaining or by immunoprecipitation followed by sequencing [8,13,14]. Additionally, sodium bisulfite-based techniques can be used to differentiate methylated and unmethylated states of DNA, as sodium bisulfite deaminates unmethylated cytosines into uracils while leaving methylated ones intact [15,16]. Combining bisulfite treatment and next-generation sequencing, therefore, allows for DNA methylation profiling across the entire genome with single-base resolution and in a quantitative manner [17,18,19,20,21].

With the advent of methylome profiling techniques, the spatiotemporal dynamics of DNA methylation during embryogenesis and development have been revealed to usually exist in cell type-specific patterns [22,23,24,25,26]. Accumulating evidence also implicates the role of DNA methylation in brain functions as well as in brain disorders, such as drug addiction [27,28,29,30,31]. Nucleus accumbens (NAc) is a key structure of the ventral striatum that plays a critical role in adaptive, goal-directed behaviors and mediates the seeking and using of addictive drugs [32,33]. In NAc, over 95% of neuronal cells are GABAergic medium spiny neurons (MSNs), which are further classified as D1 dopamine receptor-expressing MSNs (D1-MSNs) and D2 dopamine receptor-expressing MSNs (D2-MSNs) [34,35]. Furthermore, NAc D1-MSNs and D2-MSNs belong to separate circuitries that have distinct roles in drug-induced behavioral responses, whereby stimulation of D1-MSNs promotes reward and motivational behaviors, while activation of D2-MSNs inhibits these behaviors. Due to the heterogeneity of neuronal populations within NAc, it is of interest to profile the accumbal DNA methylome in a neuronal subtype-specific manner.

A major challenge to study the DNA methylome of neuronal subtypes from discrete brain regions is the limited yield of genomic DNA that can be retrieved from these cells, which is usually below the range of conventional whole-genome methylome profiling protocols. To address this, several innovative variations of these protocols have been developed, such as tagmentation-based whole-genome bisulfite sequencing [36], post-bisulfite adaptor tagging (PBAT) [25,37,38,39], and other methods using DNA modification enzymatic conversion [40,41,42]. Unlike traditional sequencing library preparations, in which substantial sample loss occurs at multiple library preparation steps, tagmentation-based methods (e.g., Tn5mC-seq) utilize a Tn5 transposase derivative to fragment DNA and append adaptors in a single step to increase library preparation efficiency. Therefore, Tn5mC-seq can be used to generate high-quality bisulfite sequencing libraries from low-input (1–10 ng) genomic DNA [36]. However, it may have potential biases due to the preference of transposase, and substantial DNA damage owing to bisulfite treatment. Traditionally, whole-genome bisulfite sequencing requires larger quantities (microgram) of DNA. This is mainly because sequencing adaptors are ligated to DNA fragments before bisulfite treatment, which results in a significant DNA template loss after bisulfite conversion. To overcome this limitation, post-bisulfite library preparation (e.g., PBAT) takes an alternative approach to prepare DNA adaptor tagging after bisulfite treatment [37]. It also incorporates random priming with biotin-labeled primers followed by enrichment of biotinylated fragments to circumvent a potential amplification failure due to bisulfite treatment-induced DNA break. However, this highly efficient low-input protocol still relies on the recovery rate of bisulfite-treated DNA and has shown GC content-related biases associated with random priming amplifications. Lately, methods based on an enzymatic conversion of methylated DNA, which do not include any bisulfite treatment, have been used to bypass the associated bisulfite-induced DNA degradation, and promise better DNA recovery than bisulfite-based approaches [40,41,42]. For example, TET-assisted pyridine borane sequencing (TAPS) adapts sequential pyridine borane reduction followed by TET1 oxidation of methylated cytosine to detect cytosine methylation of samples as little as 10 ng DNA. While these approaches have been successfully applied for low-input DNA samples in various studies, comparisons of performance and potential biases of such methods in low-abundance neuronal samples remain limited.

Here, we generated the methylome of D2-MSNs from NAc of male mice using two independent library preparation methods. The preparation was achieved through two distinct strategies from separate commercial kits: the sodium bisulfite-based Accel-NGS Methyl-Seq (AM-seq) of Swift biosciences (Cat# 30024) and the enzymatic conversion-based method Enzymatic Methyl-seq (EM-seq) from New England Biolabs (Cat# E7120S). AM-seq takes advantage of a proprietary adaptase to effectively capture single-stranded DNA and may improve library preparation efficiency after sodium bisulfite treatment of DNA. In contrast, EM-seq potentially improves the metrics of methylation profiling by substituting bisulfite treatment with enzymatic reactions; TET2 and β-glucosyltransferase are used to catalyze the conversion of methylated cytosine, followed by apolipoprotein B mRNA editing enzyme catalytic subunit 3A (APOBEC3A)-mediated deamination of unmethylated cytosines [42].

We applied fluorescence-activated cell sorting (FACS) to isolate D2-MSNs from NAc of Drd2a-eGFP (D2-eGFP) mice, in which D2-MSNs are labelled with eGFP [43]. Using 40 ng DNA available from NAc D2-MSNs of a single mouse brain, we were able to generate high-quality whole-genome methylome data using either of the two aforementioned methods. Therefore, our study not only provides valuable technical evaluations of the current methods available for low cell number whole genome methylome profiling, but also generates a reference methylome dataset of NAc D2-MSNs, which play important roles in addiction and various other neuropsychiatric disorders.

## 2. Materials and Methods

### 2.1. Animal, D2-MSN Isolation, and DNA Extraction

Male D2-eGFP mice [43] were born and raised in a controlled laboratory environment under a 12 h reverse light/dark lighting cycle at 22–25 °C. Pups were weaned at postnatal day 21 and were group-housed alongside up to 4 mice per cage with ad libitum access to food and water. All mice used in this study were of a C56BL/6J genetic background. All experiments followed the guidelines of the Animal Care and Use Committee of Florida State University.

Mouse brains were immediately removed after cervical dislocation and decapitation, and then sectioned in a stainless-steel brain matrix (1 mm intervals) on ice. Four micro-punches of NAc were collected from each brain by a 14-gauge blunt-tip needle and minced into small pieces in ice-cold Hibernate A minus calcium medium (Brainbits, #HACA500, Springfield, IL, USA) plus B27 supplement (Fisher scientific, #17504044, Waltham, MA, USA). The minced tissue was then incubated in Hibernate A minus calcium medium with 20 unit/mL papain (Worthington, #LK003176, Lakewood, NJ, USA), 5% trehalose (Acros organics, #AC30987, Geel, Belgium), and 0.1 mg/mL DNase (Worthington, #LK003170, Lakewood, NJ, USA) at 37 °C for 30 min with 700 rpm agitation. After being washed by Hibernate A minus calcium medium with 1% BSA, the tissue was triturated to cell suspension. After passing through a 70 μm cell strainer, the cell suspension was sorted on a FACS Aria SORP (BD, San Jose, CA, USA) using a 100 μM nozzle, and the GFP-positive D2-MSNs were collected in ice-cold 1× PBS buffer with 1% BSA. To isolate D2-MSNs by FACS, all cells were initially gated on SSC-A vs. FSC-A plot (‘P1’ gate). GFP positive cells represented 4–5% of the parent population (Figure S1a). Next, we derived gate ‘P6’ by plotting SSC-H vs. FSC-H, which was then used to discriminate GFP positive cells from negative cells. The GFP-positive population was increased to 9.9–12.7%, or more than 2-fold higher when plotted by just SSC-A and FSC-A. Within each sample, the P6 gating was compared to P1 gating and adjusted to match the GFP positive cell number. In total, 12k–22k GFP-positive cells were collected as D2-MSNs from each mouse brain. Using real-time PCR, we confirmed that the GFP-positive population we collected expressed high levels of a D2-MSN marker gene rather than D1-MSN marker genes (Figure S1b).

Genomic DNA was extracted with the AllPrep DNA/RNA Micro Kit (Qiagen, #80284, Hilden, Nordrhein-Westfalen, Germany) by following the product manual. Briefly, sorted cells were pelleted by 500× *g* centrifugation for 5 min, and the supernatant was carefully removed. Next, 350 μL RLT Plus buffer with 2-Mercaptoethanol added was used to release genomic DNA, which was subsequently enriched on supplied binding columns. The columns were washed with 500 μL buffer AW1, followed by 13,000× *g* centrifugation for 1 min. After another round of washing with 500 μL buffer AW2 and 13,000× *g* centrifugation for 1 min, the flow-through was removed, and the column was centrifuged at 13,000× *g* for another 2 min. DNA was then eluted into 30 μL elution buffer. In this study, three adult male mice were used: one was 96 days old and used as replicate 1 (Rep1); the other two were 97 days old and combined as replicate 2 (Rep2).

### 2.2. qPCR

Four NAc micropunches each from three untreated D2-eGFP adult male mice were used to verify the identity of FACS-isolated cells. For each sample, GFP-positive cells identified by FACS were sorted into 1 mL ice-cold TRIzol Reagent (Invitrogen, #15596026, Waltham, MA, USA), and total RNA was extracted according to manufacturer’s instructions. Briefly, cells were lysed in TRIzol Reagent, and cellular contents were separated into phases by vigorously mixing with chloroform and centrifuging. RNA was then precipitated by transferring the aqueous phase into absolute ethanol, pelleted by centrifugation, and washed with 75% ethanol. Following the final ethanol wash, RNA pellets were air-dried and resuspended in nuclease-free water. Total RNA from GFP-positive cells was reverse-transcribed into cDNA using qScript cDNA SuperMix (Quantabio, #95048025, Beverly, MA, USA). cDNA and target gene primers were mixed with PerfeCTa SYBR Green SuperMix (Quantabio, #95056500, Beverly, MA, USA) according to the manufacturer’s instructions, and target gene expression was quantified by qPCR using a 7500 Fast Real-Time PCR System (Applied Biosystems, #4351107, Waltham, MA, USA ). For every gene target, three biological replicates with two technical replicates each were used for quantification. Average target gene expression was normalized to average Gapdh expression within each sample.

The primers are as follows. Gapdh F: GGGTGTGAACCACGAGAAAT, Gapdh R: GTCTTCTGGGTGGCAGTGAT; Drd1 F: GTCTCCCAGATCGGGCATT, Drd1 R: AGTCACTTTTCGGGGATGCT; Pdyn F: TGAATCTTGGATCGGCCACC, Pdyn R: CCACGCAGATCTCAAAGCCT; Zfp521 F: GGGCCTTGCTTCCATTTTCC, Zfp521 R: TTGAGGGATCTCGGTTTCGC; Drd2 F: ATCTCTTGCCCACTGCTCTTTGGA, Drd2 R: ATAGACCAGCAGGGTGACGATGAA.

### 2.3. Sequencing Library Preparation

Each of the two biological replicates (Rep1, Rep2) were split into two 40 ng genomic DNA aliquots for AM-seq and EM-seq library preparation, respectively. For AM-seq preparation, we used the Accel-NGS Methyl-Seq DNA Library Kit (Swift, #30024, Ann Arbor, MI, USA) by following kit instructions. For EM-seq preparation, we applied the NEBNext Enzymatic Methyl-seq Kit (NEB, #E7120S, Ipswich, MA, USA) by following the product manual. Therefore, for each of the two biological replicates (Rep1, Rep2), one AM-seq and one EM-seq libraries were prepared.

For AM-seq library preparation, 40 ng D2-MSN DNA with 4 pg unmethylated lambda DNA (Promega, #D1521, Madison, WI, USA) and 4 pg CpG methylated puc19 DNA (NEB, #E7120S, Ipswich, MA, USA) as spike-in controls were fragmented to an average size of 350 bp using Covaris E220 (Covaris, Woburn, MA, USA) (60s duration, 30 peak power, 20% duty factor, 50 cycles/burst, average power 6), followed by bisulfite treatment with EZ DNA Methylation-Gold Kits (Zymo, #D5005, Irvine, CA, USA) per the provided instruction. The 20 ul fragmented DNA went through conversion with 130 μL CT Conversion Reagent by incubating for 10 min at 98 °C followed by 2.5 h at 64 °C. Each converted sample was mixed with 600 μL M-Binding buffer, loaded into an IC column, and centrifuged at 13,000× *g* for 30 s. Each column was then washed with 100 μL M-Wash buffer, followed by 13,000× *g* centrifugation for 30 s. Next, 200 μL of M-Desulphonation buffer was added to the column and incubated at room temperature for 15 min. After desulphonation, each column was centrifuged at 13,000× *g* for 30 s, washed with 200 μL M-Wash buffer twice, then 15 μL M-Elution buffer was applied to elute DNA. The resultant converted DNA was used for library preparation with the Accel-NGS Methyl-Seq DNA Library kit by following the manufacturer’s instructions. Briefly, 15 μL converted DNA was denatured for 2 min at 95 °C and then placed on ice for 2 min. Then, 25 μL Adaptase mix was added to the denatured DNA and incubated for 15 minutes at 37 °C, followed by 2 min at 95 °C. Next, 44 μL of extension reaction mix was added, and the extension reaction was carried out in a thermocycler with an extension program of 98 °C for 1 min, 62 °C for 2 min, 65 °C for 5 min, and held at 4 °C. The DNA was cleaned up with 1.2× volume of SPRIselect magnetic beads (Beckman Coulter, #B23317, Indianapolis, IN, USA) and reclaimed with 15 μL elution buffer. The ligation was then performed by adding 15 μL ligation mix followed by a purification step with 1× volume of magnetic beads. The purified DNA was then used for 7 cycles of indexing PCR with indexed primers. Finally, the amplified library was purified by using 0.85× volume of magnetic beads.

For EM-seq library preparation, 40 ng D2-MSN DNA with 4 pg unmethylated lambda DNA (Promega #D1521, Madison, WI, USA) and 4 pg CpG methylated puc19 DNA (NEB, #E7120S, Ipswich, MA, USA) as spike-in was fragmented to an average size of 270 bp using Covaris E220 (Covaris, Woburn, MA, USA) (120 s duration, 50 peak power, 25% duty factor, 1000 cycles/burst, avg power 12.5). The library was then prepared using the NEBNext Enzymatic Methyl-seq kit per the manufacturer’s instructions. For each sample, 50 μL fragmented DNA was repaired with 10 μL End Prep Mix at 20 °C for 30 min and followed by incubation at 65 °C for 30 min. Repaired DNA was ligated with methylated adaptors, purified with 110 μL magnetic beads and reclaimed with 28 μL elution buffer. The 28 μL purified DNA was used for methylcytosine oxidation with 17 μL TET2 reaction mix and 5 μL Fe(II) solution. After 1 h of TET2 oxidation at 37 °C, the reaction tube was placed on ice, 1 μL of Stop Reagent was added, and samples were incubated at 37 °C for 30 min to stop the oxidation reaction. The oxidated DNA was purified with 90 μL magnetic beads and eluted in 16 μL elution buffer. Then, 4 μL 0.1 M NaOH was added to denature the purified oxidated DNA at 50 °C for 10 min, and the reaction tube was placed on ice after the denaturation. Deamination was immediately carried out by adding 80 μL APOBEC reaction mix and incubated at 37 °C for 3 h. The converted DNA was purified with an equal volume of magnetic beads and reclaimed with 20 μL elution buffer. Indexed primers were added to purified DNA for 5 cycles of amplification, and each amplified library was purified with 0.9× volume of magnetic beads.

The AM-seq and EM-seq libraries were assayed by a 4200 TapeStation system (Agilent, Santa Clara, CA, USA), and the molar concentrations were quantified by a KAPA library quantification kit (Roche #07960336001, Basel, Switzerland). The libraries were then pooled with base-balanced libraries and sequenced for 100 bp paired-end reads on Illumina Novaseq 6000 S1 flow cell with 30% spike-in of whole-genome sequencing libraries. All libraries were sequenced twice and combined to reach the final read depths.

### 2.4. AM-Seq and EM-Seq Data Analysis 

FASTQ files containing sequencing reads were checked by FastQC v0.11.9 (https://www.bioinformatics.babraham.ac.uk/projects/fastqc/, accessed on 31 January 2020). To remove low-quality sequences (Quality Phred score < 20) and artificial adaptor sequences, Trim Galore 0.6.4 and Cutadapt 1.18 were used. Due to the synthetic adaptors generated during library preparation, we trimmed an additional 18 bp from the 5’ end and 4 bp from the 3’ end for both reads of AM-seq libraries. For both reads of EM-seq libraries, we trimmed 8 bp from the 5’ end and 4 bp from the 3’ end according to the positional base content bias result by FastQC. Read-pairs with any one read less than 20 bp were removed. Processed read-pairs were aligned to the male mm10 mouse genome (https://www.encodeproject.org/files/male.mm10, accessed on 9 February 2019) by Bismark [44]. We performed alignment with the same processed reads as well as DNA reference sequence of lambda DNA or puc19 DNA to assess the conversion rate. After alignment, deduplication, and filtration of unconverted read pairs, methylation sites with genomic base coordinates and genomic context information were extracted by Bismark_methylation_extractor. Downstream analysis was performed with methylation sites separated according to their dinucleotide context (CG, CA, CT, CC).

### 2.5. Bioinformatic Analysis of Methodology Comparison

We used CollectGcBiasMetrics of Picard (http://broadinstitute.github.io/picard/, accessed on 18 August 2021) and QualiMap v.2.2.2-dev [45] to perform coverage analysis. For unconverted tetranucleotide bias analysis, the frequency of unconverted tetranucleotides was extracted by Bismark. For coverage deviation per dinucleotide analysis, 4 million randomly sampled aligned read pairs (~1% of total) were analyzed against randomly shuffled regions in the covered genomic region of the same library, and this analysis was repeated three times with different sampling seeds. All called methylation sites were used to calculate the global CpG and CpH methylation levels.

The genomic features of CpG islands were downloaded from the University of California at Santa Cruz (UCSC) Table Browser [46]. A list of putative mouse striatum enhancers was obtained from EnhancerAtlas 2.0 [47], and liftOver was applied to convert enhancer coordinate annotations in mm9 to mm10. Transcription starting sites (TSS) and other gene annotations were extracted from the Ensembl gene annotation system (Mus_musculus.GRCm38.90) [48]. The aggregated methylation profile was generated using BEDTools [49] and Deeptools [50]. To calculate methylated sites, we performed a binomial test as previously reported [51]. The non-conversion rate for each cytosine context was used to calculate the binomial *P*-value, and a threshold was chosen with false discovery rate (FDR) less than 1%.

### 2.6. Unmethylated Region Analysis

The unmethylated regions (UMRs) were identified by MethylSeekR with recommended parameters (30 CpG sites, 50% methylation level threshold) [52,53] using CpG methylation data after merging the four individual D2-MSN methylomes. The genomic annotation of UMR was performed by “annotatePeaks.pl” of Homer [54]. Histone ChIP-seq data was retrieved with the accession number GSE42810 and GSE63749 from the National Center for Biotechnology Information (NCBI) Sequence Read Archive (SRA). Motif enrichment was conducted by “findMotifsGenome.pl” of Homer. The genomic data files were transformed to bigWig format and visualized by IGV 2.8.6.06 [55]. The GO analysis of UMR regions was performed in R with the “clusterProfiler” package [56].

### 2.7. Differential Methylation Region (DMR) Analysis

The D2-MSN methylome was compared to the methylomes of mouse PFC CamKIIa-positive (CamKIIa+) excitatory neurons (GSM1541958 and GSM1541959) [22] and neural progenitor cells (GSE111283) [57]. Methylome data of MethylC-seq were downloaded from NCBI. We filtered the common mouse SNP sites and removed sites with coverage less than 4. To perform differential methylation analysis for CpG methylation, DSS [58] with a smoothing function [59] was used to identify DMRs. The methylation change threshold was set as 20%, and the *P*-value threshold was set as 1× 10^−5^ to focus on substantially changed regions. We merged DMRs within 500 bp and set the length threshold for CpG DMR as 100 bp with a minimum of 5 CpG sites.

For non-CpG methylation, we chose to analyze methylated CpA dinucleotides (mCpA) as a representative [60]. In calculating mCpA levels of all genes, only the longest isoform of each gene was used. The methylation level of 1 kb windows with 500 bp slide was used for differential mCpA analysis with DSS, the methylation change threshold was set as 2%, and the *P*-value threshold as 0.01. We merged mCpA DMRs within 50 kb and set the length threshold as 2 kb. The web-based tool GREAT [61] was used to perform gene ontology analysis for DMR regions.

## 3. Results

### 3.1. Whole Genome Methylome Library Preparation and Sequencing

**Figure 1:**
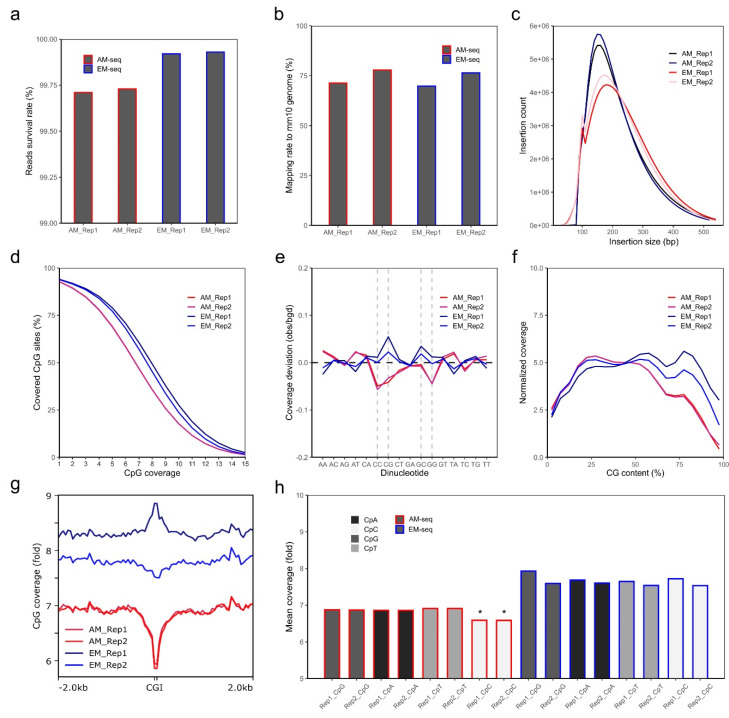
Quality analysis of NAc D2-MSN methylome libraries. (**a**) Survival rate of sequencing reads for each library. The calculation was performed after adaptor trimming, quality trimming, and short reads removal. Y-axis starts from 99%. (**b**) Unique alignment rate of each library. (**c**) Library insertion size distribution and count numbers. Data were extracted only from aligned fragments. (**d**) Cumulative coverage plot shows percentages of CG sites (out of all CG sites in the mm10 genome) reaching a certain amount of CpG coverage. Data of CpG coverage from 1- to 15-fold are shown. (**e**) Coverage deviation between AM-seq and EM-seq at each dinucleotide context. CC, CG, GC, and GG are dash-lined to highlight the divergent biases between AM-seq and EM-seq. (**f**) The normalized coverage according to CpG content percentage. (**g**) CpG coverage at around CpG islands (CGIs). Coverage data of CGI with extended 2 kb up- and down-stream regions are plotted with a 50 bp bin size. (**h**) The mean coverage at each CpN context (CpA, CpC, CpG, CpT). Note the relative lower coverage of CpC in AM-seq samples (*).

One AM-seq and one EM-seq libraries were generated from each of the two biological replicates (Rep1, Rep2) of mouse nucleus accumbens D2-MSNs. Similar amounts of DNA libraries were obtained from AM-seq (7 cycles of PCR library amplification) and EM-seq (5 cycles of PCR library amplification) (Table S1). Each library yielded around 400 M paired-end reads (Table S1). Though all reads have consistently high quality, read 1 of all libraries is of notably better quality than read 2, with fewer base pairs removed during quality-trimming (Table S2). After adaptor trimming, quality trimming (q > 20), and read length filtering, over 99.6% of all reads passed quality thresholds. AM-seq has a slightly lower survival rate than EM-seq, mainly because AM-seq has more short reads (<20 bp) that were removed (Figure 1a, Table S2).

Using an unmethylated lambda DNA spike-in, we calculated the conversion rates of CpG, CpA, CpT, and CpC dinucleotides. The CpG conversion rates of lambda DNA are over 99.8% for both AM-seq and EM-seq. EM-seq had slightly lower non-conversion rates (0.1% ~ 0.2%) when compared to AM-seq in all 4 CpN (CpG, CpA, CpT, CpC) contexts (Figure S2a). Furthermore, AM-seq libraries also had the highest non-conversion rates at CpA sites, whereas EM-seq had the lowest non-conversion rates at CpA sites (Figure S2a, Figure S2b). Using CpG-methylated puc19 DNA as a conversion control, we found that AM-seq libraries had slightly higher over-conversion rates (3–4%) compared to EM-seq (1–2%) (Figure S2c).

Trimmed sequencing reads were then aligned to the male mouse mm10 reference genome, which revealed that the mapping ratio is more variable across biological replicates than library preparation methods (Figure 1b). In either AM-seq or EM-seq, Rep 2 had ~6% higher mapping ratio than Rep 1. For each sample, the AM-seq library had slightly higher (~1%) mapping ratios than that of its EM-seq library counterpart. The duplication rates of the four libraries were between 13.5% and 17.5% (Figure S2d), and the lower duplication rates in EM-seq indicate that the EM-seq libraries had better library complexity, given that they had the same sequencing depth as AM-seq but with two fewer PCR amplification cycles during library preparation. Consistent with this finding, when we extrapolated the library sizes using Preseq [62], we found the predicted unique fragments of the four libraries were between 1.4–1.6 billion, with better complexity and larger library sizes in EM-seq (Figure S2e).

AM-seq was previously reported to have an excellent genomic coverage [63]. By comparing AM-seq and EM-seq, we found both methods generated similar genome coverage across the whole genome (Figure S2f). We achieved over 15-fold genome coverage with 400 M paired-end reads in each of the four libraries. Comparing the libraries derived from the same DNA, EM-seq yielded higher coverage compared to AM-seq. Part of the coverage difference may be attributed to the randomly added synthetic tail (~15 bp) to 3’ ends of ssDNA during AM-seq library preparation, which required extra base pair sequence trimming before alignment. Another possible explanation is that EM-seq libraries had larger insert sizes as shown in sequencing (Figure 1c) or TapeStation analysis (Figure S2g), with AM-seq libraries shifted more toward the smaller size range, whereas EM-seq libraries had a more balanced size distribution. 

We next extracted the methylated cytosine sites from the four libraries after balancing them to the same uniquely aligned reads by down-sampling. From an equal amount of uniquely aligned reads, similar coverage was observed between the two methods, with EM-seq having a slightly better performance. Out of the ~43.7 million CpG sites in the mouse genome (mm10), EM-seq covered more than 41 million (93.8% of total CpG sites) CpG sites, compared to AM-seq at about 40.5 million (92.7% of total CpG sites). EM-seq also had marginally better depth of CpG sites coverage than AM-seq (Figure 1d). Among them, the EM-seq Replicate 1 (EM_Rep1) library had the longest inserts (Figure 1c) and the highest CpG coverage after balancing aligned fragment counts.

Sodium bisulfite treatment, upon which the AM-seq protocol is based, has been known to cause DNA breaks and have biases at CG-rich regions during PCR amplification [21,64]. In comparison to the conventional whole genome bisulfite sequencing method, AM-seq was reported to perform better with respect to nucleotide amplification bias [63]. We found that both AM-seq and EM-seq have minuscule coverage deviations per dinucleotide context. However, compared to EM-seq, AM-seq had lower coverages at CpC, CpG, and GpG dinucleotides, possibly related to the bisulfite-induced DNA damage (Figure 1e). Notably, we found EM-seq had an over-representation at CpG and GpC dinucleotides, which indicates the bias might be an effect of high hydrogen bonding in CpG dinucleotides on PCR amplification, or of the TET2-mediated enzymatic conversion. We also noted a consistent trend that the genomic regions with higher CG content are less represented in AM-seq (Figure 1f). For example, at CpG islands (CGIs), which are genomic regulatory elements characterized by a high frequency of CpG sites and hypomethylation [65], AM-seq had lower (~14% less) coverage than at adjacent regions, whereas EM-seq lacked any such variation. Similarly, EM_Rep1 had an increased coverage around CGI regions, while EM_Rep2 showed a slight decrease (Figure 1g). The average coverage for single-stranded CpN sites is 6.6 and 6.9 folds for the two AM-seq libraries, respectively, and more than 7.5 folds for the two EM-seq libraries (Figure 1h). We also noticed AM-seq had a slightly lower CpC coverage than CpG, CpA, and CpT, which were more similar to each other (Figure 1h).

### 3.2. Methylation Profiling of D2-MSNs

**Figure 2:**
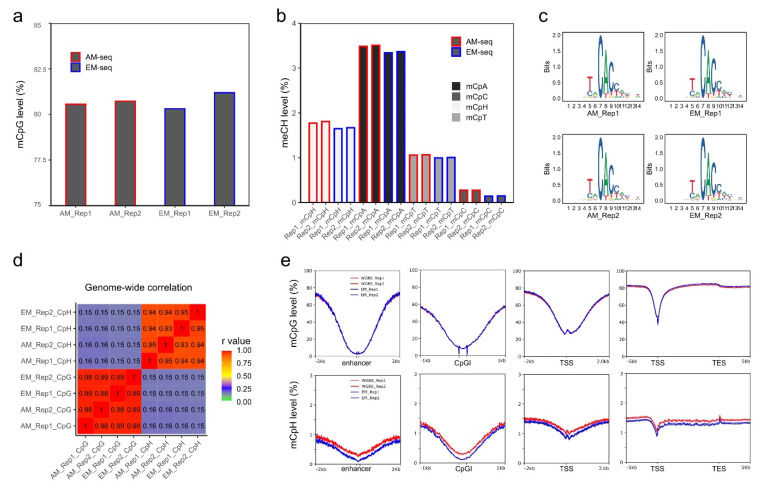
CpG and non-CpG methylation in NAc D2-MSNs. (**a**) Global CpG methylation levels calculated from AM-seq and EM-seq libraries. Y-axis starts from 75%. (**b**) Non-CpG methylation levels. mCpH represents the (methylated CpA, CpT, and CpC)/(total CpA, CpT, and CpC). (**c**) DNA motif of top hypermethylated non-CpG methylation sites. (**d**) Pearson correlation of mCpG and mCpH levels of the four methylome libraries using 10 kb bins. (**e**) Methylated CpG levels and methylated CpH levels at enhancers, CGIs, TSSs, and gene proximal regions using 50 bp bins.

Next, the global methylation levels were calculated after cytosine methylation site extractions. The four libraries had comparable global CpG methylation levels ranging from 80.3–81.2% (Figure 2a). Both AM-seq and EM-seq detected a slightly higher CpG level in Rep 2 than in Rep 1 (0.16% higher by AM-seq and 0.88% higher by EM-seq). Furthermore, the methylation level measurements between the two methods were very close, with the CpG level of EM_Rep1 being 0.2% lower than that of AM_Rep1, and the CpG level of EM_Rep2 being 0.3% higher than AM_Rep2.

In addition, for non-CpG methylation, EM-seq demonstrated consistently lower methylation levels (Figure 2b) when compared to AM-seq. The methylated CpH (mCpH) levels (measurements of mCpA, mCpC, and mCpT together) were 1.77% and 1.8% in the two libraries of AM-seq and 1.65% and 1.67% in Rep1 and Rep2 of EM-seq, respectively. In D2-MSNs, similar to the previously reported non-CpG methylation distribution in neuronal methylomes [23], mCpA was found to be the predominant methylated non-CpG. Furthermore, we noted that, in both AM-seq and EM-seq datasets, mCpG accounted for 65–67% of all cytosine methylation events, while mCpA and mCpT were responsible for ~25% and ~7%, respectively. CpC had the lowest methylation frequency, which was 1.3–1.4% in AM-seq and 0.7–0.8% in EM-seq (Figure S3a). To identify high-confidence non-CpG methylation sites, we performed a binomial test with a false discovery rate method [23] and observed closer numbers of methylated CpG sites (~52%) and methylated non-CpG sites (~48%) in AM-seq, and slightly less methylated CpG sites (47%) than methylated non-CpG sites (53%) in EM-seq (Figure S3b).

Furthermore, the mCpA sites in D2-MSNs usually have an adjacent ‘T’ upstream and a ‘CC’ dinucleotide immediately downstream (Figure 2c). This sequence motif coincides with DNMT3A binding sites and is consistent with previous neuronal methylome reports [23,24,51,66]. Interestingly, the flanking sequences (5’-T and 3’-CC) not only exist at mCpA sites, but also occur at CpT, and CpC methylation sites (Figure S3c), which indicates a functional role of flanking DNA sequences at non-CpG methylation sites.

To examine the reproducibility of D2-MSN methylomes, we calculated the correlation of 10 kb-binned mCpG and mCpH levels (Figure 2d). We found a strong correlation of mCpG methylation between the two D2-MSN replicates using either library preparation method. A significant correlation of mCpH methylation between the two biological replicates was also observed. Furthermore, we confirmed a strong correlation between methylomes generated by the two different methods (Figure 2d). As we observed subtle coverage bias between AM-seq and EM-seq according to GC content, we matched aggregated methylation levels of all four methylome libraries at various genomic regions (Figure 2e). We found that both mCpG and mCpH methylation are depleted at enhancers, CGIs, and transcription start sites (TSSs). Though the distribution patterns of CpG and CpH methylation in AM-seq and EM-seq were well-matched across these genomic features, we noticed that the CpH methylation levels of AM-seq libraries were slightly higher than that of EM-seq (Figure 2e). We then stratified CpH methylation to mCpA, mCpT, and mCpC, and confirmed their consistent methylation patterns as well as relatively lower methylation levels in EM-seq (Figure S3d).

### 3.3. D2-MSN Unmethylated Regions (UMRs)

**Figure 3:**
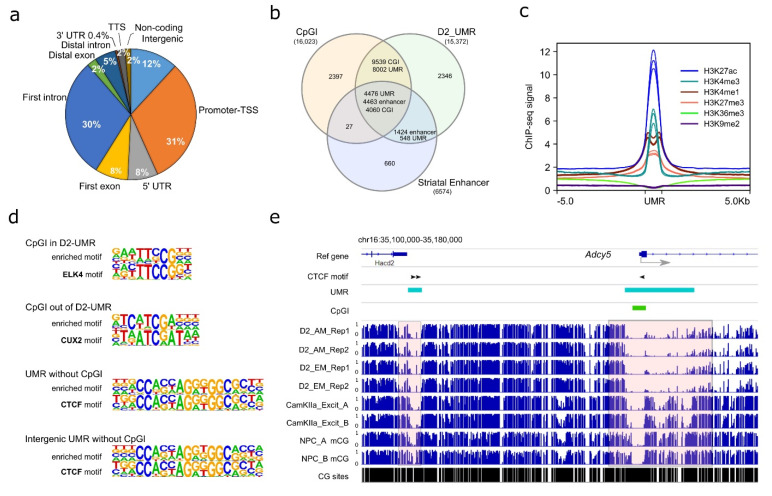
Characterization of NAc D2-MSN UMRs. (**a**) Genomic feature distribution of NAc D2-MSN UMRs. They are mostly located in TSS-proximal regions, including “Promoter-TSS”, “5’-UTR”, “First exon”, and “First intron”. (**b**) D2-MSN UMRs significantly overlap with CGIs and striatal enhancers. (**c**) Histone modification enrichments at D2-MSN UMRs. H3K4me1, H3K4me3, H3K27ac, H3K27me3, H3K9me2, H3K36me3 Chromatin IP signals are presented along up- and down-stream 5 kb regions of UMR sites. RPKM normalization is performed across all histone ChIP-seq data. (**d**) The top enriched motif sequence and matched transcription factor in each of the four categories of CGI/UMR regions. CGI in D2-UMR: CpG islands located in D2-MSN UMRs; CGI out of D2-UMR: CpG islands located outside of D2-MSN UMRs; UMR without CGI: D2-MSN UMRs that do not have a CpG island inside; intergenic UMR without CGI: intergenic D2-MSN UMRs that do not have a CpG island inside. (**e**) *Adcy5* locus with two neighboring D2-MSN UMRs. The proximal UMR overlaps with a CGI and the distal UMR does not. The two shaded areas highlight regions with cell type-specific differential methylation. D2: D2-MSNs. CamKIIa_Excit: PFC CamKIIa+ excitatory neurons. NPC: neural progenitor cells. AM: AM-seq. EM: EM-seq. CG sites lane shows the CpG frequency. Black arrowhead indicates a CTCF motif in UMR regions.

Using AM-seq and EM-seq, we profiled the D2-MSN methylome from the NAc of adult male mice. Given the high reproducibility of the four D2-MSN methylomes, we merged them to achieve a combined ~30-fold coverage for each strand to identify D2-MSN-specific regulatory regions. While the majority of the mammalian genome is highly methylated, a small portion of regions remains hypomethylated and are usually enriched with regulatory elements. Therefore, we focused on unmethylated regions (UMRs), which are long stretches of DNA segments with high CG frequency (i.e., more than 30 CpG sites per region) and low DNA methylation levels (Figure S4a) that may have regulatory roles on transcription [51,52,67,68,69,70]. We found 15,310 UMRs (Table S4), with the majority of them located at regions close to transcription start sites, such as promoters, first introns, first exons, and 5’ UTRs (Figure 3a). In addition, 12% of UMRs were located in intergenic regions. In comparison to annotated mouse CGIs (UCSC Table Browser) and reported mouse striatal enhancers [47], the majority of UMRs (85% of all UMRs) overlap with either 85% of CGIs or 89% of striatal enhancers (Figure 3b). The remaining 15% of UMRs that were not located at a CGI or enhancer may represent additional regulatory regions with low methylation levels in D2-MSNs. Furthermore, when compiled with the previously published mouse NAc histone modification ChIP-seq data [71], we found the UMRs were enriched with histone markers H3K4me1, H3K27ac, H3K4me3, and H3K27me3, which are usually associated with enhancers (H3K4me1, H3K27ac), active transcription initiation sites (H3K4me3), and repressive regulation (H3k27me3). In contrast, we found no enrichment of H3K36me3 or H3K9me2 at UMR regions (Figure 3c).

To gather biological insight of D2-MSN UMRs, we then performed de novo motif analysis at four genomic feature regions: 1. “CGI in UMR” (CpG islands located in D2-MSN UMRs), 2. “CpG out of UMR” (CpG islands located outside of D2-MSN UMRs), 3. “UMR without CGI” (D2-MSN UMRs that do not have a CpG island inside), or 4. “Intergenic UMR without CGI” (intergenic D2-MSN UMRs that do not have a CpG island inside) (Table S5). The enriched motifs in the four categories of regions were matched to dozens of DNA binding proteins (Table S6). For example, the highest ranked motifs for CGIs in UMRs (i.e., low methylation level at these CGIs) belonged to the ELK of the ETS family. In CGIs out of UMR, the top motif is CUX2, indicating accumulation of DNA methylation silencing around those regions in D2-MSNs (Figure 3d). The most significantly enriched motif in UMRs without CGI was matched to CTCF [72], and CTCF motif enrichment was also ranked first in intergenic UMRs without CGI (Figure 3d), which suggests an interplay between DNA methylation and higher order chromosomal organization in D2-MSNs. A representative example is shown near *Adcy5* (Figure 3e), a gene in which mutation is associated with various brain disorders [73]. This gene has one upstream distal UMR, which does not overlap with a CGI, and another UMR that runs across the TSS and does overlap with a CGI. Two CTCF binding sites were predicted in the distal UMR, and one such motif existed in the proximal UMR. In comparison to published methylome datasets from mouse neural progenitor cells (NPCs) [57] and mouse prefrontal cortex (PFC) CamKIIa+ excitatory neurons [22], we found both UMRs have differential methylation patterns among the three cell types, with robust hypomethylation in D2-MSNs. Therefore, such UMR-specific methylation changes demonstrate methylation dynamics that are specific to D2-MSNs.

Furthermore, we performed pathway analyses of these four categories of UMR related genomic regions in D2-MSNs [74]. We found that the 13,550 genomic regions in the “CGIs within UMR” category covered a broad range of genes and biological pathways (Figure S4b, Table S7), whereas the other three categories were enriched with limited numbers of specific pathways. “CGIs outside UMR” were mainly associated with genes in the “Hippo signaling pathway” and “Signaling pathways regulating pluripotency of stem cells” pathways, which is consistent with the notion that DNA methylation represses pluripotency genes in mature neurons. Genes associated with “UMR without CGI” also had pertinent pathways enriched, such as “oxytocin signaling pathway”, “glutamatergic synapse”, and “long-term depression”. Interestingly, the GO term “alcoholism” was enriched in genes associated with the “CGI-free intergenic UMR” category, including *Shc3*, *Shc4*, *Araf*, *Fosb*, *Hdac11*, *Adcy5*, *Creb3l2*, *Gnai2*, *Grin2d*, and *Ppp1r1b*. This suggests a regulatory role of intergenic region D2-MSN-specific DNA methylation in alcohol addiction.

### 3.4. D2-MSN Specific CpG Differential Methylation Regions (DMRs)

**Figure 4:**
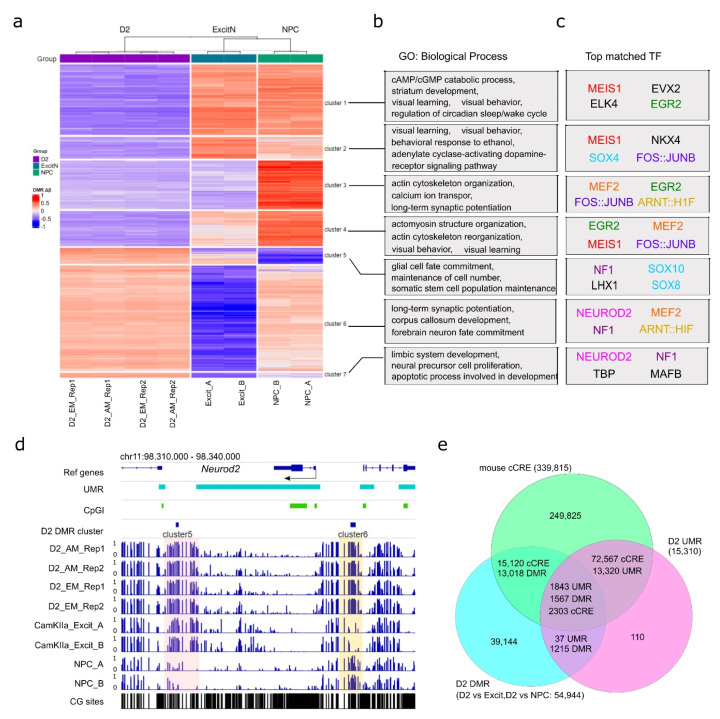
NAc D2-MSN-specific CpG DMRs. (**a**) K-means clustering of 51,704 NAc D2-MSN-specific autosomal CpG DMRs, in comparison to NPCs and PFC CamKIIa+ excitatory neurons. Two sets of DMRs are combined. For each DMR, the delta β values of individual samples (compared to the average methylation level across all samples) are used for clustering. (**b**) Representative biological process gene ontology (GO) terms enriched with genes associated with each cluster of CpG DMRs in panel a. (**c**) Transcription factors (TFs) with matched sequences of the top 4 motifs enriched in each of the seven CpG DMR clusters. Identical or similar transcription factors are in the same color, except where black is used for motifs only recognized in a single cluster. (**d**) The genomic locus of *Neurod2* is a representative of transcription factor genes enriched in D2-MSN hyper CpG DMRs with D2-MSN-specific hypermethylation. The shaded regions indicate notable cell type-specific differential methylation when compared to NPCs or PFC excitatory neurons. (**e**) UMRs and cell type-specific DMRs are largely overlapped with mouse cCREs. D2: D2-MSNs. CamKIIa_Excit: PFC CamKIIa+ excitatory neurons. NPC: neural progenitor cells. AM: AM-seq. EM: EM-seq.

To further explore D2-MSN regulatory regions beyond UMRs, we performed differential CpG methylation analysis by comparing our data to the methylomes of postnatal day 3–4 mouse NPCs [57], and CamKIIa+ excitatory neurons from PFC of 8–14 weeks old adult male mice [22]. Both datasets were generated using the MethylC-seq protocol [75] with around seven-fold coverage of each strand. Compared to accumbal D2-MSNs, the global CpG methylation level was about 2% lower in NPCs (~78.5%) and slightly higher (less than 1%) in excitatory neurons (81.2% and 81.4% for two replicates). Furthermore, we identified 24,284 CpG DMRs between D2-MSN and NPC methylomes, with the majority of them (21,854) being hypomethylated in D2-MSNs (Figure 4a, Table S8), supporting the concept of accumulation of global methylation during cell differentiation with widespread depletion of methylation at regulatory regions [57,76,77]. When compared to PFC excitatory neurons, we identified 30,660 CpG DMRs with 10,440 hypomethylatedDMRs and 20,220 hypermethylatedDMRs (Figure 4a, Table S9) in D2-MSNs. Most of these CpG DMRs were located within distal introns and intergenic regions (Figure S5a).

We then performed a K-means clustering analysis to classify all autosomal CpG DMRs into seven clusters (Figure 4a, Table S10). We observed that the four libraries were better clustered together by biological replicates than by methods. This was further confirmed by PCA analysis (Figure S5b). Based on the methylation status in comparison to PFC excitatory neurons and NPCs, the D2-MSN hypomethylation CpG DMRs were classified into four clusters (clusters 1 to 4), while the D2-MSN hypermethylation CpG DMRs were separated into three clusters (clusters 5 to 7) (Figure 4a). CpG DMRs of clusters 1, 2, and 4 represent regions where CpG methylation is significantly depleted in D2-MSNs compared to the other two cell types. DMRs in cluster 3 show reduced methylation levels in both D2-MSNs and PFC excitatory neurons when compared to NPCs. Though fewer in number, the cluster 5 CpG DMRs represent genomic regions that gained CpG methylation in both NAc D2-MSNs and PFC excitatory neurons. DMRs in cluster 6 represent divergent hypermethylation in D2-MSNs and hypomethylation in PFC excitatory neurons stemming from moderate DNA methylation in NPCs. Lastly, cluster 7 shows regions with contrasting hypermethylation in D2-MSNs and hypomethylation in PFC neurons and NPCs (Figure 4a). Using ontology analysis, we found each of the seven DMR clusters was enriched with a defined set of biological process terms (Figure 4b). Generally, genes associated with hypomethylation in NAc D2-MSNs (e.g., clusters 1, 2, 3, and 4) were mostly enriched with various terms related to neuronal functions (Figure 4b, Table S11), such as “cAMP/cGMP catabolic process”, “dopamine receptor signaling”, “ion transport”, “learning”, and “behavior”. In contrast, genes associated with hypermethylated DMRs in both NAc D2-MSNs and PFC excitatory neurons (cluster 5) were enriched with terms related to “maintenance of cell number”, “glial cell fate commitment”, and “somatic stem cell population maintenance”. In addition, genes associated with PFC-specific hypomethylated DMRs (clusters 6, 7) demonstrated enrichments in “long-term synaptic potentiation”, “forebrain neuron fate commitment”, “limbic system development”, and “apoptotic process involved in development”. Together, this suggests that D2-MSN-specific DNA methylation changes may play dual roles in both cell fate commitment during development and biological function specification.

Next, we carried out a motif analysis to identify the transcription factors that may be associated with methylome remodeling (Figure 4c, Table S12). We found that each of the seven clusters was enriched with a unique set of transcription factor binding motifs. Among them, AP-1 transcription complex (FOSB::JUNB) motifs were one of the top hits in clusters 2, 3, and 4. Motifs of EGR2, MEF2, MEIS1, and NF1 were also enriched in three clusters. Notably, AP-1 is a heterodimer of the FOS family and JUN family and has been recognized as a key transcription factor in addiction and stress response [78,79,80]. In particular, FOS family genes are transiently induced in NAc by acute drug exposure, with a truncated form of FOSB (ΔFOSB) demonstrating a lasting accumulation [32,81]. The immediate early genes EGR2 and EGR1 have also been implicated in drug response and may be subjected to drug-induced DNA methylation changes as well [82,83,84]. Similarly, MEF2 in NAc has also been reported to regulate synapse plasticity and sensitized responses to cocaine [85]. The enrichment of these motifs in D2-MSN CpG DMRs not only supports the functional roles of the relevant transcription factors in drug addiction but also implies a D2-MSN-specific function that is DNA methylation dependent.

In addition, we recognized that some motif enriched transcription factors themselves were subjected to DNA methylation changes. For example, the NEUROD2 motif was highly enriched in clusters 6 and 7 of D2-MSN DMRs (Figure 4c), which was accompanied by two hypermethylated DMRs near the *Neurod2* gene (Figure 4d); the upstream DMR was heavily methylated in NAc D2-MSNs but had moderate methylation in NPCs and low methylation in PFC excitatory neurons, while the downstream DMR showed hypermethylation in both neuronal cell types.

By comparing the methylation-related regulatory regions that we identified as UMRs and CpG DMRs with the curated set of mouse candidate *cis*-regulatory elements (cCREs) from the ENCODE project [86], we found that almost all UMRs overlapped with one or more cCRE. Out of the 15,310 UMRs, 15,163 overlapped with 74,870 cCREs, and 14,585 D2-MSN CpG DMRs overlapped with 17,423 cCREs (Figure 4e). Furthermore, a total of 1567 D2 CpG DMRs were located in 1843 UMR regions covering 2303 cCREs. This further supports the regulatory role of DNA methylation in cell identity and function in NAc D2-MSNs.

### 3.5. D2-MSN non-CpG DMRs

We then analyzed non-CpG methylation in NAc D2-MSNs by comparing them to NPC and PFC excitatory neurons, respectively. Mammalian non-CpG methylation accumulates during development, remains prevalent in neurons, and is inversely correlated with gene transcription [23,87,88]. Given that the methylomes of NPCs and excitatory neurons were produced by a different protocol (MethylC-seq), we only considered CpH sites that passed the binomial test in order to mitigate any potential bias that may be derived from methylation profiling methodologies (Figure 2b). We thus confirmed the non-CpG methylation differences among cell types. The NPC methylome had a significantly lower level of global mCpH (less than 0.001%) compared to NAc D2-MSNs and the PFC excitatory neurons, which were both ~1%. As mCpA accounts for the majority of non-CpG methylation, which also highly correlates with total mCpH methylation, we analyzed CpA methylation as a representative of non-CpG methylation. We first calculated the gene body CpA methylation levels of all genes. By overlapping the top 1000 (~5% of all genes) highly methylated genes in D2-MSNs and PFC excitatory neurons, we found that 349 genes obtained high levels of non-CpG methylation in both cell types. Among them, genes encoding microRNA, transcription factors, and peptide ligands were over-represented (Table S13).

**Figure 5:**
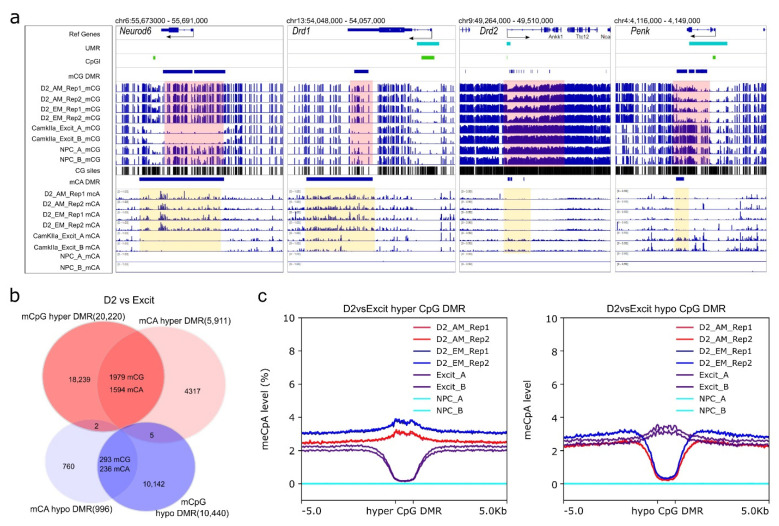
NAc D2-MSN-specific non-CpG DMRs. All non-CpG DMRs are identified by comparing NAc D2-MSNs to PFC CamKIIa+ excitatory neurons (D2 vs. Excit). (**a**) Representative view of *Drd1* and *Neurod6* loci with CpG and CpA hypermethylation in D2-MSNs. In contrast, D2-MSN marker genes *Drd2* and *Penk* have CpG and CpA hypomethylation along their genomic loci when compared to PFC CamKIIa+ excitatory neurons. Pink-shaded regions highlight CpG DMR regions, yellow-shaded regions highlight CpA DMR regions. β value range of all CpG lanes is 0–1. β value range of CpA for *Neurod6* and *Drd1* is 0–1. β value range of CpA for *Drd2* and *Penk* is 0–0.5. NPC has virtually no CpA methylation. (**b**) CpG DMRs and CpA DMRs are significantly overlapped. (**c**) CpA methylation levels at CpG DMRs. Left: CpA methylation levels of D2 hyper-methylated CpG DMRs (blue and red) are noticeably higher than surrounding regions in D2-MSNs and much lower in PFC excitatory neurons (purple). Right: The CpA methylation levels of D2 hypo-methylated CpG DMRs are much lower in D2-MSNs (blue and red) and are discernibly higher in PFC excitatory neurons(purple) compared to the adjacent regions. The NPC methylome is devoid of non-CpG methylation (cyan).

Next, we performed differential analysis with binned CpA methylation windows to identify non-CpG DMRs (996 hypomethylated DMRs, and 5911 hypermethylated DMRs in comparison to PFC CamKIIa+ neurons) (Table S14). We found that the genes associated with hypomethylated non-CpG DMRs were specifically clustered in the ontology categories related to D2-MSN functions, such as “gamma-aminobutyric acid (GABA) biosynthetic process”, “gamma-aminobutyric acid (GABA) metabolic process”, “learning or memory”, and “dopamine receptor signaling pathway” (Figure S6a, Table S15). In contrast, genes associated with hyper non-CpG DMRs were enriched in GO terms mostly related to development or differentiation (Table S15). Given the repressive role of non-CpG methylation on transcription, this suggests these genes were silenced during D2-MSN maturation. For example, dopamine receptor *Drd1* and transcription factor *Neurod6*, which are not expressed in mature D2-MSNs, were found to have hyper non-CpG DMRs (Figure 5a). To further support the functional role of CpA methylation in D2-MSNs, we found non-CpG hypo DMRs in the gene body of two D2-MSN marker genes: *Drd2* and *Penk*. These non-CpG DMRs also appeared to overlap with CpG DMR in the same trend (Figure 5a). Further analysis of all CpG DMRs with non-CpG DMRs demonstrated substantial overlaps between CpG hyper-DMRs (~9.8% overlaps) with mCpA hyper-DMRs (~27.0% overlaps), and mCpG hypo-DMRs (~2.8% overlaps) with mCpA hypo-DMRs (~23.7% overlaps) (Figure 5b). By analyzing the mCpA levels at all CpG DMR regions, we found that PFC excitatory neurons had significantly decreased CpA methylation at CpG DMRs that were hypermethylated in D2-MSNs, and D2-MSNs hypomethylated DMRs also had significant CpA hypomethylation (Figure 5c). In light of the observed synergy, we performed a regression analysis between CpG methylation and non-CpG methylation levels at CpG DMRs. The results presented a moderate correlation (0.475–0.645) between the two modalities in CpG DMRs. The CpG methylation can explain ~ 42% variance of CpA methylation in excitatory neurons, and 22–25% variance in D2-MSNs (Figure S6b). 

## 4. Discussion

In this study, we characterized the mouse NAc D2-MSN methylomes using two different methods, namely AM-seq and EM-seq, using the Swift Accel-NGS Methyl-Seq DNA Library Kit and the NEB Enzymatic Methyl-seq Kit, respectively. Our side-by-side comparison demonstrates good consistency and reproducibility between the two methods. We found the library quality and yields, global methylation and loci specific methylation, and potential biases were all highly comparable between the two methods. AM-seq or EM-seq was compared to other methods, such as MethyC-seq [89], QIAseq [63], and PBAT [90], with better performance. However, as both methods were suitable to handle lower amounts of DNA (ng scale), a direct comparison between the two will be helpful when deciding on a method for whole-genome methylome profiling with low cell numbers, such as neuronal subtypes from discrete brain regions. In studies with brain tissue, methylation landscapes of different neural cell types are usually superimposed, which inevitably obscures epigenetic changes that are often cell type-specific, given the remarkable neuronal heterogeneity in the brain. Therefore, brain cell type-specific methylation profiling is necessary to aid our understanding of the functional role of DNA methylation in the brain. Using D2-MSNs from mouse nucleus accumbens as an example, our study shows that these two methods are comparable and suitable for methylome profiling of low-input DNA samples (i.e., 40 ng DNA extracted from D2-MSNs isolated from NAc of a single mouse). We found the CpG methylation levels to be closely correlated not only between biological replicates, but also between the two methods. A similar correlation also holds for non-CpG methylation. Notably, both AM-seq and EM-seq detected the subtle difference in global CpG methylation levels between Rep 1 and Rep 2. Moreover, D2-MSN CpG DMRs were slightly more separated according to biological replicates than methods in the first principal component of PCA analysis, indicating that methylation variations between individual mice were similarly detected by both methods. However, it should be noted that the methylomes generated from the two methods were not identical. For example, the library yield of EM-seq was substantially higher than AM-seq, considering EM-seq took two fewer PCR amplification cycles to generate similar amounts of libraries from AM-seq. The library yield with less amplification cycles is beneficial for library complexity, which is also supported by our analysis. In addition, we found that EM-seq libraries had a larger insert size, which should contribute to better data yield and mapping ratio, and generated more valid reads under the same expense. While optimization is possible for both methods, we have followed the default library preparation protocols and sonicated the gDNA into the recommended fragmentation sizes (350 bp for AM-seq, 270 bp for EM-seq). The eventual shorter library inserts in AM-seq could be attributed to the DNA destruction under sodium bisulfite treatment. Moreover, AM-seq had a slightly higher unique mapping ratio and over-conversion rate of mCpG, while the two protocols exhibited different dinucleotide preferences of non-conversion and coverage bias per GC content. Despite the subtle individual differences between the two biological replicates, we have observed good consistency among our methylome datasets. A recent study reported the single-cell methylome profiling of the mouse cortex, which identified 161 cell clusters including D1-MSNs and D2-MSNs based upon the methylation status of marker genes [91]. Our datasets provide a valuable reference with a different cell isolation strategy and high genomic coverage. In addition, we need to point out that neither of the two methods we applied in this study can distinguish methylated cytosine from its oxidative derivatives. As sodium bisulfite does not convert 5-hydroxymethylcytosine (5hmC), 5hmC cannot be separated from 5-methylcytosine (5mC) in AM-seq. Furthermore, the methylated cytosine callings in EM-seq may not be distinguished from 5hmC, 5-formylcytosine (5fC), nor 5-carboxylcytosine (5caC) [40]. Since 5hmC is most abundant in the brain [23,92,93,94], it would be beneficial to chart 5hmC at base-resolution in MSNs in the future. Though 5fC and 5caC are only present at extremely low levels [95], whether they contributed to the methylation differences between AM-seq and EM-seq deserves further investigation.

The gain of non-CpG methylation in neuronal cells is known to have a repressive effect on gene expression, possibly through MECP2 [23,88,96,97,98,99,100]. Notably, we found that the hyper- and hypo- non-CpG methylation regions in D2-MSNs appear to be enriched with contrasting functionally distinct gene categories. Additionally, the selective deposition and depletion of non-CpG methylation are in accordance with the notion that non-CpG methylation may serve as a fine-tuning mechanism in brain function. We found a significant overlap between D2-MSN CpG DMRs and non-CpG DMRs, with a moderate correlation between CpG and non-CpG methylation levels in those CpG DMR regions. This noticeable synergy might result from shared gene transcriptional activity or other local epigenetic modalities.

In NAc, the two types of principal neurons, D1-MSNs and D2-MSNs, belong to distinct neural circuits [35,101,102]. It has long been recognized that D1- and D2-MSNs not only have unique molecular signatures that mediate their respective functions [103,104], but also play contrasting roles in behaviors. For example, activation of D2-MSNs inhibits reward and motivational behaviors [105,106,107], whereas activation of D1-MSNs promotes the responses. Notably, the NAc neuronal methylome was recently reported to be more distinct compared to the ones from other brain regions, with the differential methylation regions being highly enriched for heritability of addictive behaviors [60,108]. The DNA modification enzymes were also subjected to expression changes in NAc after cocaine exposure, which may alter neural plasticity and genome-wide DNA modification landscapes [109,110]. With such accumulating evidence, to study neuron subtype-specific roles of DNA epigenetics in NAc medium spiny neurons becomes necessary. Through the characterization of NAc D2-MSN methylome, and its comparison to NPCs and PFC excitatory neurons, we identified numerous regulatory regions that include 15,310 UMRs and 54,944 D2-MSN-specific CpG DMRs. Almost all UMRs and about one-fourth of D2-MSN CpG DMRs overlapped with at least one cCRE curated by the ENCODE project, which indicates the important role of DNA methylation in D2-MSN functions. For example, the motif analysis of UMRs lacking a CGI has shown an enrichment of CTCF binding. CTCF plays pivotal roles in higher-order genome architecture [72,111] that may mediate a broad range of cellular functions [112,113]. The DNA binding affinity of CTCF was found to be negatively affected by DNA methylation [114,115,116]. Crystal structure analysis [117] and direct binding assays [118,119] further supported that CpG methylation inhibits the DNA binding affinity of CTCF. Recently, growing evidence indicates the role of chromatin architecture in brain disorders, such as addiction [120,121,122,123]. For example, cocaine exposure was found to increase DNA methylation, decrease CTCF binding, and inhibit the three-dimensional looping interaction between the *Auts2* and *Caln1* genes in mouse nucleus accumbens, which was associated with D2-MSN-specific upregulation of *Auts2* [124]. In addition, a smoking-related DNA methylation change was found near the CTCF locus in postmortem NAc tissue [125]. Our identification of CTCF motif enrichment in NAc D2-MSN-specific UMRs and the recognition of an “alcoholism” pathway within the same UMR category suggests D2-MSN-specific interplay between DNA methylation and higher order chromatin architecture in addiction [122].

## 5. Conclusions

Taken together, we performed a comparison of two methylome profiling methods using 40 ng DNA from mouse NAc D2-MSNs. Though the two methods are based upon distinct chemistries, the methylome data generated from them were highly comparable. We therefore provided a valuable resource of the neuron subtype-specific methylome of mouse nucleus accumbens D2-MSNs. 

## Data Availability

The raw reads files and processed data were deposited to the NCBI Gene Expression Omnibus with accession number GSE195752.

## Supplementary Materials

The following supporting information can be downloaded at: https://www.mdpi.com/article/10.3390/genes13020306/s1. Figure S1: D2-MSN FACS plots and qPCR validation, Figure S2: D2-MSN AM-seq and EM-seq quality analysis, Figure S3: NAc D2-MSN methylome characterization, Figure S4: D2-MSN UMR analysis, Figure S5: NAc D2-MSN-specific CpG DMRs, Figure S6: NAc D2-MSN non-CpG methylation, Table S1: DNA input and yield of D2-MSN methylomes, Table S2: Sequencing reads processing, Table S3: Statistics of the four D2-MSN methylomes, Table S4: D2-MSN unmethylated regions (UMRs), Table S5: Four categories of CGI/UMR related genomic regions, Table S6: Transcription factor motifs enriched in CGI/UMR regions, Table S7: List of KEGG pathways enriched in genes associated with four categories of CGI/UMR regions, Table S8: CpG DMRs of D2-MSNs vs. NPCs, Table S9: CpG DMRs of D2-MSNs vs. PFC CamKIIa+ neurons. Table S10: D2-MSN autosomal CpG DMRs used for K-means clustering analysis, Table S11: List of enriched GO terms for seven clusters of D2-MSN CpG DMRs, Table S12: Transcription factors matched by D2-MSN CpG DMR motif analysis, Table S13. Genes with high non-CpG methylation in both D2-MSNs and PFC excitatory neurons, Table S14: Non-CpG methylation DMRs comparing D2-MSNs to PFC CamKIIa+ neurons, Table S15: List of enriched GO terms for D2-MSN non-CpG DMRs.
