# Supplementary material for: Whole Genome DNA Methylation Profiling of D2 Medium Spiny Neurons in Mouse Nucleus Accumbens Using Two Independent Library Preparation Methods
Source: Genes (Basel). 2022 Feb 6;13(2):306. doi: 10.3390/genes13020306 (PMC8872013; doi:10.3390/genes13020306)
Supplement: Supplementary file 1 [file genes-13-00306-s001.zip › Sup.figures.pdf]

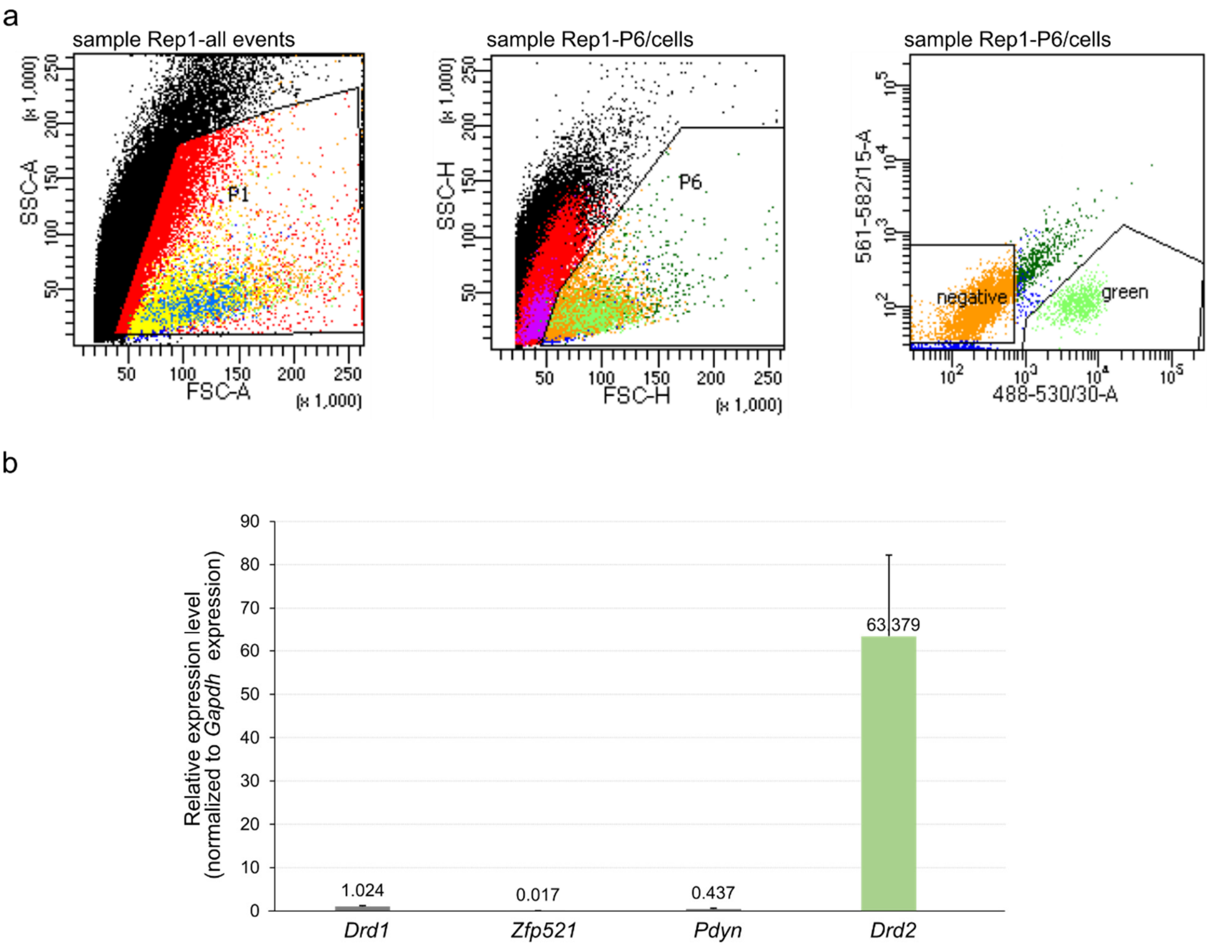

Figure S1. D2-MSN FACS plots and qPCR validation. (a) FACS plots of sample "Rep1". Left: P1 gate of FSC-A vs SSC-A out of all events. Middle: P6 gate of FSC-H vs SSC-H. Right: cells in P6 were replotted by fluorescence signal and green (GFP-positive) D2-MSNs were collected. (b) Relative gene expression of D1-MSN-specific (*Drd1*, *Zfp521*, and *Pdyn)* and D2-MSN-specific (*Drd2)* genes in FACS-isolated NAc D2-MSNs. Gene expression is normalized to *Gapdh*. N=3 biological replicates.

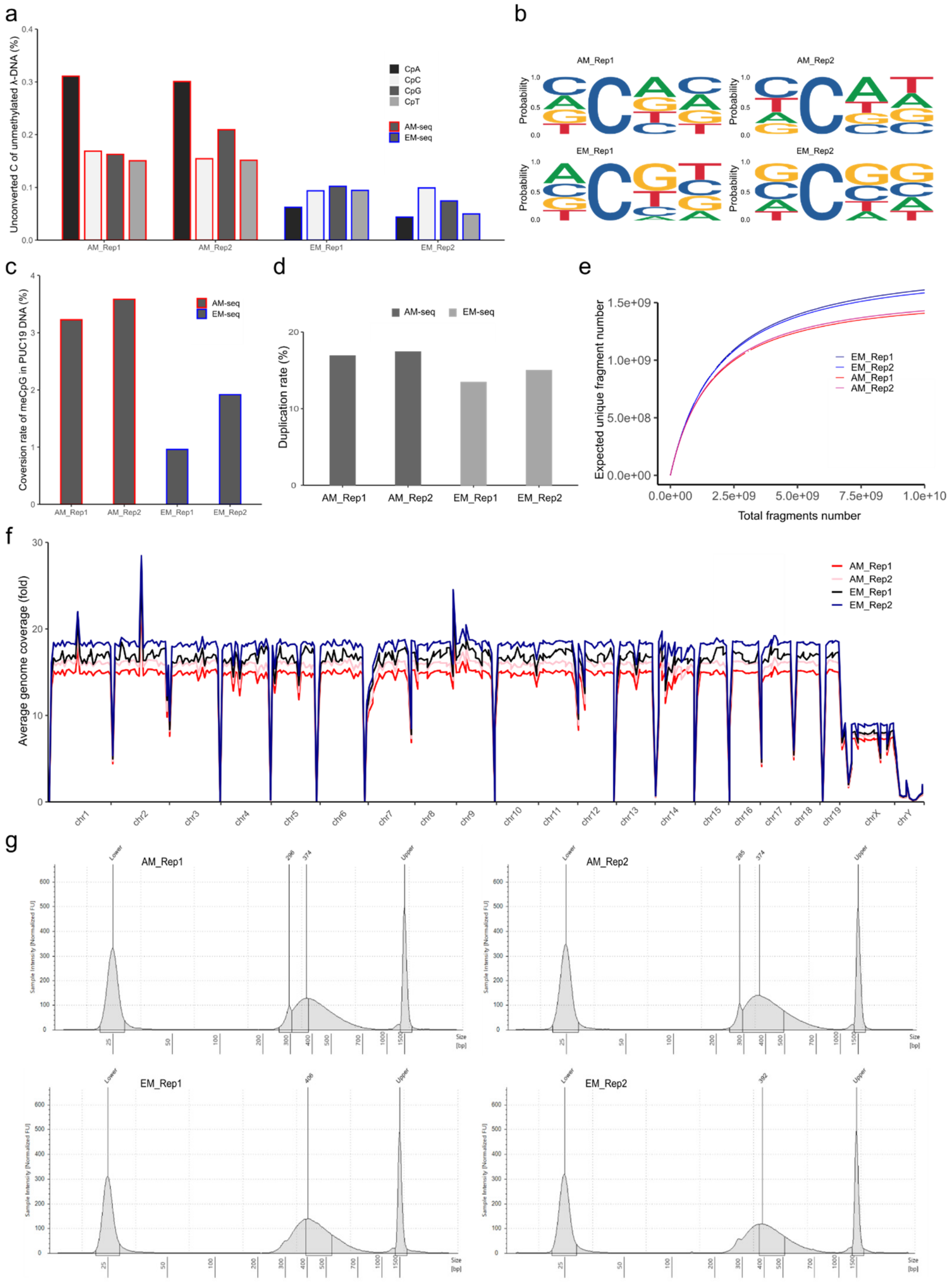

Figure S2. D2-MSN AM-seq and EM-seq quality analysis. (a) Cytosine un-conversion rates of unmethylated control DNA (spike-in lambda DNA) at different cytosine contexts. (b) Base frequency of unconverted sites in AM-seq and EM-seq. DNA bases shown on top are of higher frequency. Analysis was derived from spike-in unmethylated lambda DNA. (c) Conversion rates of methylated CpG sites in control spike-in puc19 DNA. (d) Sequencing duplication rate of each library. (e) Library size of unique fragments. (f) Genomic coverage of AM-seq and EM-seq libraries in each chromosome. (g) Library size distribution plots by TapeStation assay.

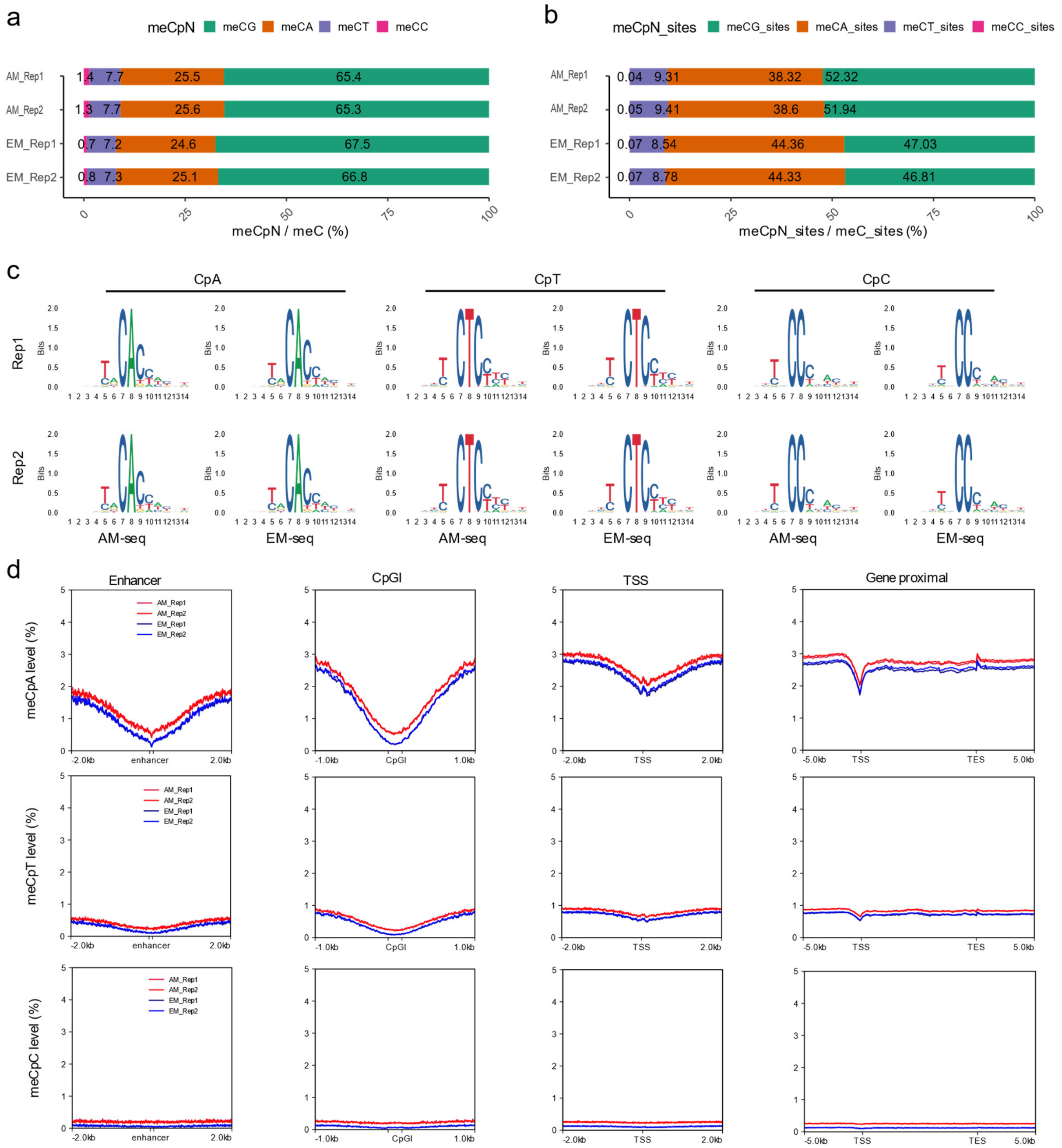

Figure S3. NAc D2-MSN methylome characterization. (a) Percentage of each methyl-CpN (CpA, CpC, CpG, CpT) events out of total methylated-cytosines. (b) Percentage of each methyl-CpN (CpA, CpC, CpG, CpT) sites that have passed the binomial test with a false discovery rate method. (c) Enriched DNA motif based upon the top 10,000 hypermethylated CpA, CpT, or CpC sites. (d) Methylation levels of CpA, CpT, and CpC at enhancers, CGIs, TSSs, and gene proximal regions. A bin size of 50 bp was used for motif analysis. AM-seq and EM-seq were analyzed separately in all characterizations.

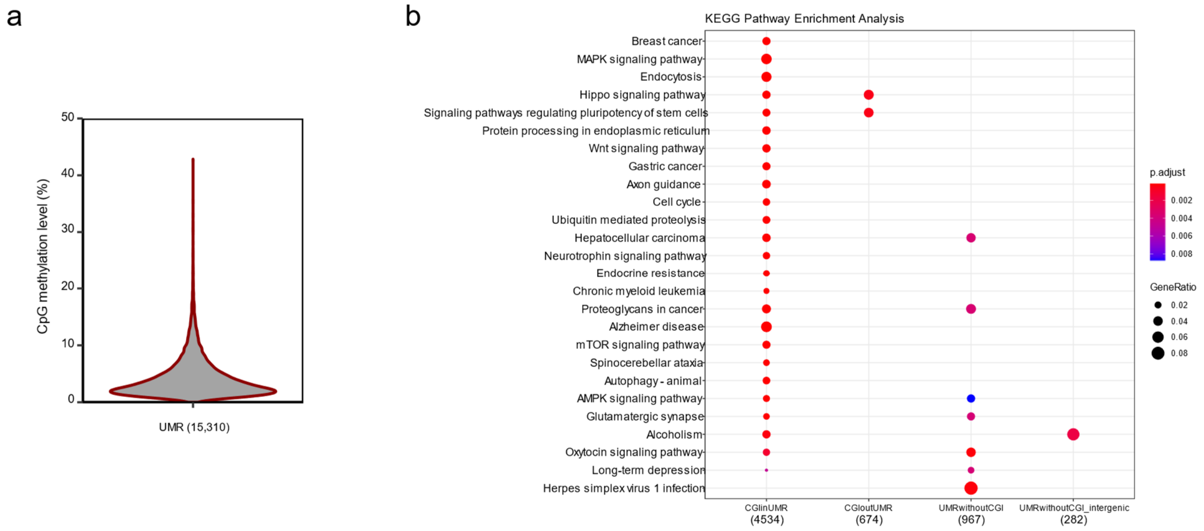

Figure S4. D2-MSN UMR analysis. (a) Violin plot shows methylation level distribution of all D2-MSN UMRs (N=15,310). (b) KEGG pathway enrichment analysis of four CGI/UMR related genomic regions in NAc D2-MSNs. CGI in UMR: CpG islands overlapped with D2-MSN UMRs; CGI out UMR: CpG islands located outside of D2-MSN UMRs; UMR without CGI: D2-MSN UMRs without a CpG island inside; UMR without CGI Intergenic: Intergenic D2-MSN UMRs without a CpG island inside.

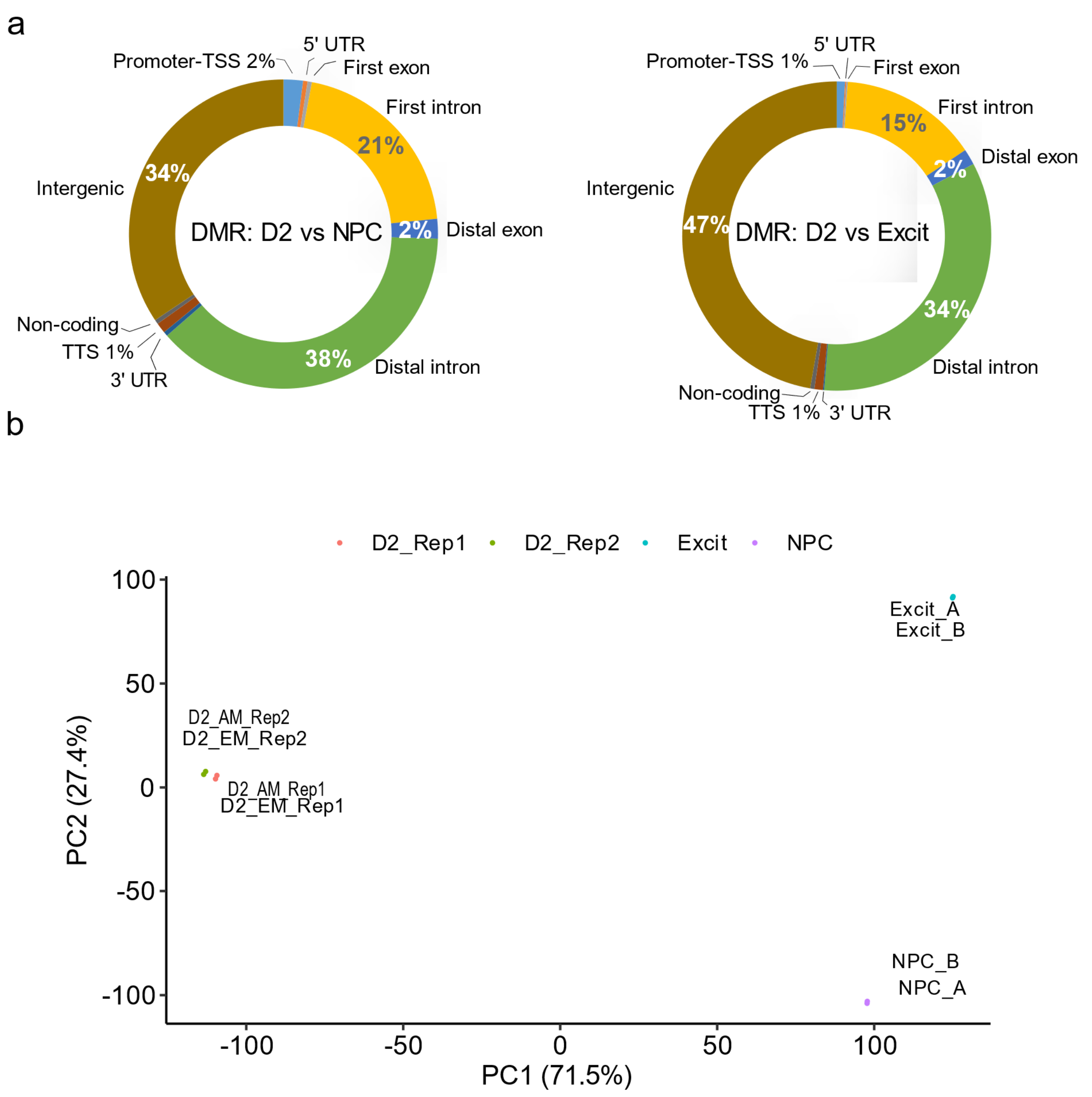

Figure S5. NAc D2-MSN-specific CpG DMRs. (a) Genomic distribution of D2-MSN CpG DMRs in comparison to neural progenitor cells (NPC) (left), or PFC CamKIIa+ excitatory neurons (Excit) (right). (b) PCA analysis of NAc D2-MSNs, NPCs, and PFC excitatory neurons using CpG methylation levels of CpG-DMRs. The “variance explained” by PC1 is 71.5%, and the “variance explained” by PC2 is 27.4%.

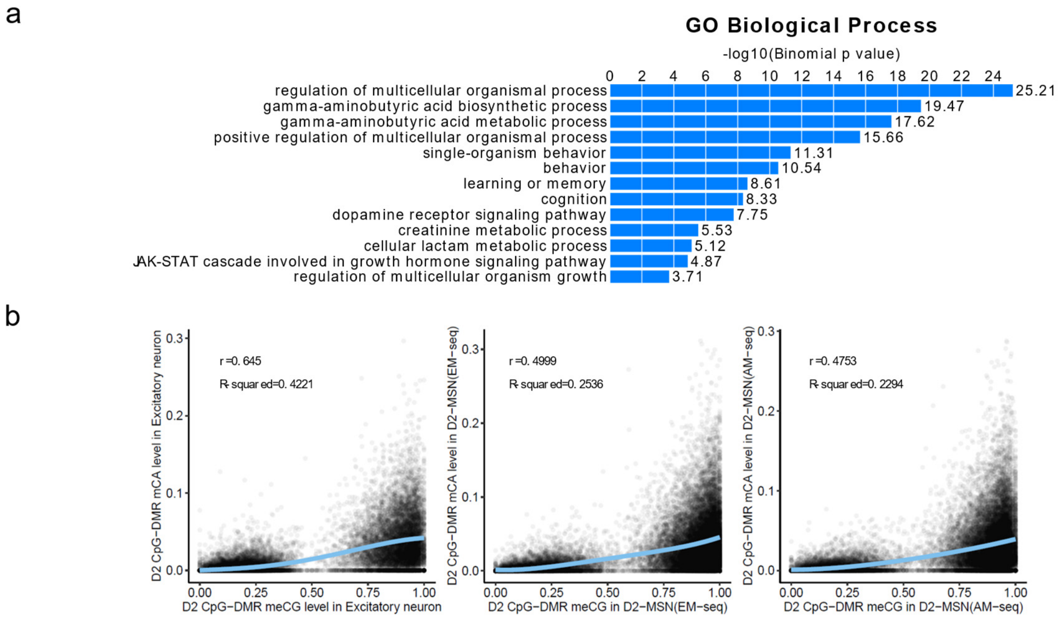

Figure S6. NAc D2-MSN non-CpG methylation. (a) Biological Process Gene Ontology (GO) analysis of hypomethylated CpA DMRs. (b) Synergy between mCpA and mCpG. Regression analysis using mCpA and mCpG levels in PFC excitatory neurons (left), in NAc D2-MSNs (by EM-seq) (middle), and in NAc D2-MSNs (by AM-seq) (right). Correlation coefficient r is calculated with Pearson correlation. R-squared is calculated after a linear regression modeling in R.
